# Anterior Minithoracotomy: a Safe Approach for Surgical ASD Closure & ASD Device Retrieval

**DOI:** 10.21470/1678-9741-2017-0024

**Published:** 2017

**Authors:** Vivek Wadhawa, Chirag Doshi, Manish Hinduja, Pankaj Garg, Kartik Patel, Amit Mishra, Pratik Shah

**Affiliations:** 1 Department of Cardiovascular and Thoracic Surgery of the U. N. Mehta Institute of Cardiology and Research Center (affiliated to BJ Medical College, Ahmedabad), Gujarat, India; 2 Department of Research of the U. N. Mehta Institute of Cardiology and Research Center (affiliated to BJ Medical College, Ahmedabad), Gujarat, India

**Keywords:** Heart Septal Defects, Atrial/Surgery, Sternotomy, Minimally Invasive Surgical Procedures

## Abstract

**Objective:**

Midline sternotomy is the preferred approach for device migration following transcatheter device closure of *ostium secundum* atrial septal defect. Results of patients operated for device migration were retrospectively reviewed after transcatheter closure of atrial septal defect.

**Methods:**

Among the 643 patients who underwent atrial septal defect with closure device, 15 (2.3%) patients were referred for device retrieval and surgical closure of atrial septal defect. Twelve patients underwent device retrieval and surgical closure of atrial septal defect through right antero-lateral minithoracotomy with femoral cannulation. Three patients were operated through midline sternotomy.

**Results:**

Twelve patients operated through minithoracotomy did not require conversion to sternotomy. Due to device migration to site of difficult access through thoracotomy, cardiac tamponade and hemodynamic instability, respectively, three patients were operated through midline sternotomy. Mean aortic cross-clamp time and cardiopulmonary bypass time were 28.1±17.7 and 58.3±20.4 minutes, respectively. No patient had surgical complication or mortality. Mean intensive care unit and hospital stay were 1.6±0.5 days and 7.1±2.2 days, respectively. Postoperative echocardiography confirmed absence of any residual defect and ventricular dysfunction. In a mean follow-up period of six months, no mortality was observed. All patients were in New York Heart Association class I without wound or vascular complication.

**Conclusion:**

Minithoracotomy with femoral cannulation for cardiopulmonary bypass is a safe-approach for selected group of patients with device migration following transcatheter device closure of atrial septal defect without increasing the risk of cardiac, vascular or neurological complications and with good cosmetic and surgical results.

**Table:** 

Abbreviations, acronyms & symbols	
ASD	= Atrial septal defect
CPB	= Cardiopulmonary bypass
LPA	= Left pulmonary artery
LVOT	= Left ventricular outflow tract
TEE	= Transesophageal echocardiography

## INTRODUCTION

*Ostium secundum* atrial septal defect (ASD) is one of the most common congenital anomalies. Presently, transcatheter device closure is a preferred technique for the management of ASD and usually patients who are not suitable for transcatheter closure are referred for surgical closure^[1]^. Device malposition and migration are the major complications following transcatheter management, however, they are infrequent and occur in 1.1% to 3.5% of patients^[2-4]^. Device migration may be life-threatening and requires emergent or urgent surgical intervention.

Minimally invasive congenital cardiac surgery is a developing field^[5]^. Presently, mini-invasive approach is preferred for elective surgical closure of ASD as it provides the cosmetic advantage and avoids sternal complications^[6-8]^. However, patients who develop complications due to transcatheter management are preferably operated through midline sternotomy due to the apprehension of worsening a complicated condition. Therefore, the patients who develop a complication following transcatheter management typically lose the advantage of mini-invasive approach.

At our institute, we managed selected patients with complication following transcatheter ASD closure through mini-invasive approach. In this article, our technique and results are retrospectively reviewed.

## METHODS

### Patients

Between January 2012 and June 2016, 643 patients underwent transcatheter device closure of ASD at our institute. Among them, 15 (2.3%) patients were referred for urgent device retrieval and surgical ASD closure due to device migration. The hospital data of these 15 patients were retrospectively reviewed for demographic and operative details of the patients, postoperative morbidity and outcome. The study was approved by our institutional ethics committee (UNMICRC/CVTS/18) and consent from the patients was waived off in view of retrospective nature of the study.

### Diagnosis and Surgical Management

The diagnosis of device migration was confirmed by echocardiography. All the patients were operated within one hour after confirmation of device migration. Among 15 patients, 12 patients were operated through right anterolateral thoracotomy mini-invasive approach with femoral arterial and venous cannulation for establishment of cardiopulmonary bypass (CPB) while three patients underwent classical median sternotomy.

Exclusion criteria for anterior minithoracotomy were:

1. Hemodynamic instability
2. Device migration to left ventricular outflow tract (LVOT), aortic and distal PA

**Fig. 1:**
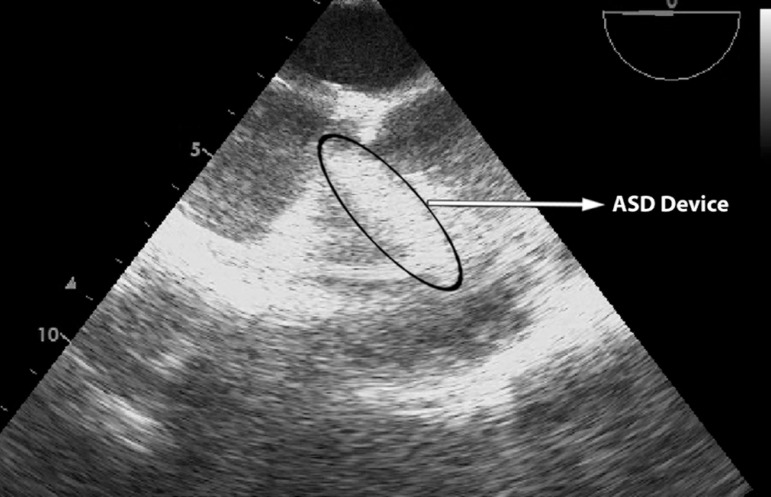
Mid oesophagus 0º Four Chamber view showing ASD device in right ventricular outflow tract.

In all the patients, device retrieval and ASD surgical closure was done. All the patients were operated in hybrid catheterization lab where the transcatheter procedure was performed. Intra operative transesophageal echocardiography (TEE) was performed in all the patients (Figure 1). All the patients were operated by a single surgeon.

### Surgical Technique of Anterior Minithoracotomy

#### Anesthetic protocol

The induction of anesthesia included midazolam (0.1 mg/kg) and fentanyl (5 µg/kg). Muscle relaxation was achieved by pancuronium (0.1 mg/kg). All the patients received single lumen cuffed endotracheal intubation. Fentanyl (total dose of 15 to 20 µg/kg) with the addition of sevoflurane was used for maintenance. After the induction of anesthesia, external defibrillation pads were placed. Invasive monitoring included right radial artery catheter and a triple-lumen central venous catheter.

#### Patient position

Patients were laid supine with bolster under right scapula to raise right hemithorax at 30º. The arms were positioned beside the body, the right groin was prepared and draped to allow access to the right femoral vessels.

#### Surgery

**Fig. 2:**
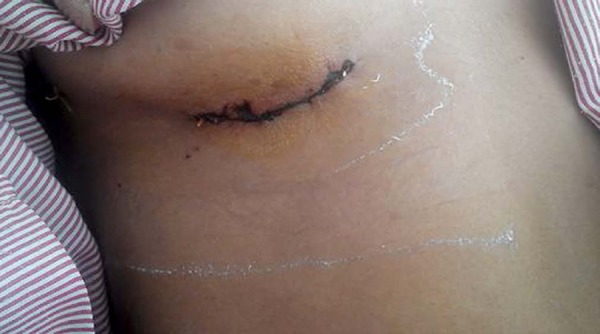
Postoperative aspect of the minithoracotomy incision.

**Fig. 3:**
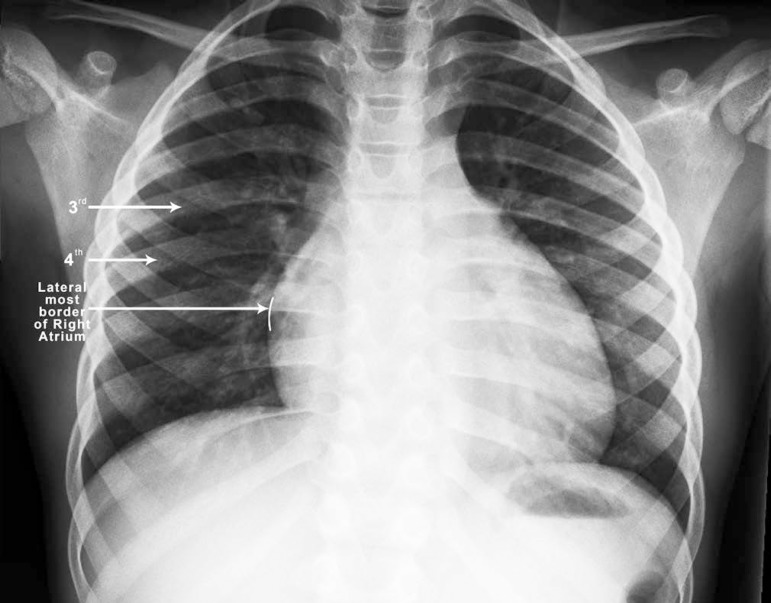
Chest X-ray with posteroanterior (PA) view showing the lateral border of the right atrium in the 4^th^ intercostal space. The intercostal space for access to the pleural cavity was decided on the basis of the anterior intercostal space overlying the lateral border of the right atrium.

The skin incision 4-6 cm length was made along the right inframammary groove between the parasternal and midaxillary lines (Figure 2). The breast tissue and pectoralis major muscle were dissected *en bloc* from the chest wall. Right pleural cavity was entered through the right anterior 3^rd^, 4^th^ or 5^th^ intercostal space according to the chest X-ray. The intercostal space for access to the pleural cavity was decided on the basis of the anterior intercostal space overlying the lateral most border of right atrium (Figure 3). Using Seldinger's technique femoral artery was cannulated (Medtronic, DLP, Femoral Artery Cannula) and for venous cannulation a dual stage venous cannula (Medtronic, CARPENTIER, Bi-caval femoral cannula) was inserted through a small transverse incision at the inguinal region (opposite to that utilized for device placement). CPB was instituted and maintained with mild hypothermia (32ºC). The lung was retracted posteriorly and the pericardium was then opened longitudinally 3-4 cm anterior to the phrenic nerve extending the pericardiotomy towards aorta and downwards to the diaphragm. Pericardial stay sutures were put on traction to elevate the mediastinal structures and move right atrium in direct view.

The dual stage femoral venous cannula was directed into SVC under TEE guidance. SVC and IVC were snugged. The cannulation of ascending aorta, for cardioplegia (MiAR^TM^ Cannulae, Medtronic, USA), was done with downward traction of right atrium by a simple vascular clamp. Minimally invasive instruments (Fehling Instruments, Germany) were used, although simple instruments are enough. The ascending aorta was cross clamped with a Chitwood type transthoracic aortic clamp through the third intercostal space in the mid-axillary line. This incision could be used for later chest tube insertion.

Myocardial protection was achieved with antegrade cardioplegia in aortic root, then the right atrium was opened and stay sutures were used for better exposure. After examination of cardiac anatomy, the device was retrieved and the defect closed with gore-tex/pericardial patch or directly depending on the size and shape of ASD. The right atrium was closed in double layers and deairing was done with the aortic root vent connected to suction. The absence of intracardiac air and the quality of repair were evaluated by TEE. Sinus rhythm was restored. The CPB was gradually discontinued. After discontinuation of CPB, de-cannulation, administration of protamine, hemostasis, and chest tube insertion was done. Before closing the chest, an intercostal block was performed. The chest was then closed in a routine fashion with an intradermal continuous suture for the skin.

## Statistical Analysis

Normally distributed continuous data was expressed as mean ± S.D, whereas non-normal data was depicted as median with range. All data analysis was performed using SPSS for windows, version 20.0 (SPSS Inc., Chicago, IL, USA).

## RESULTS

Fifteen patients (10 females) with age 30.67±16.72 (range 5 to 65 years) and weight 43.07±13.78 (range 13 to 65 kg) were operated for device retrieval and surgical closure of ASD (Table 1). Twelve patients were operated through minithoracotomy while three underwent classical median sternotomy.

**Table 1:** Preoperative demographic and clinical data.

Variable	Hospital A (N=106)	Hospital B (N=508)
Age at surgery	6 m (4 d - 16 y)	17 m (0 d - 66 y)
Gender (male)	60 (56%)	271 (53%)
Weight (kg)	5.15 (0.65-59.55)	9.20 (1.00-102.00)
Weight-for-age Z-score < -2	40 (37.7%)	161 (37.1%)
Length or height (cm)	62 (32-159)	79 (36-183)
BMI (kg/m^2^)	13.9 (6.5-24.3)	15.6 (6.8-37.5)
BMI-for-age Z-score < -2	29 (27.4%)	101 (23.3%)
Prenatal diagnosis	8 (9.2%)	64 (16.6%)
Number of previous surgeries	0 (0-5)	0 (0-5)
Prematurity	21 (19.8%)	32 (6.3%)
Major noncardiac structural anomaly	0	3 (0.6%)
Combination procedure	12 (11.3%)	28 (5.5%)
Preoperative hematocrit (%)	35 (23-63)	40 (12-65)
Preoperative SaO_2_ (%)	93 (45-100)	96 (29-100)

BMI=body mass index; SaO_2_=arterial oxygen saturation; m=months; d=days; y=years Values are expressed as median (range) or count (proportion).

**Fig. 4:**
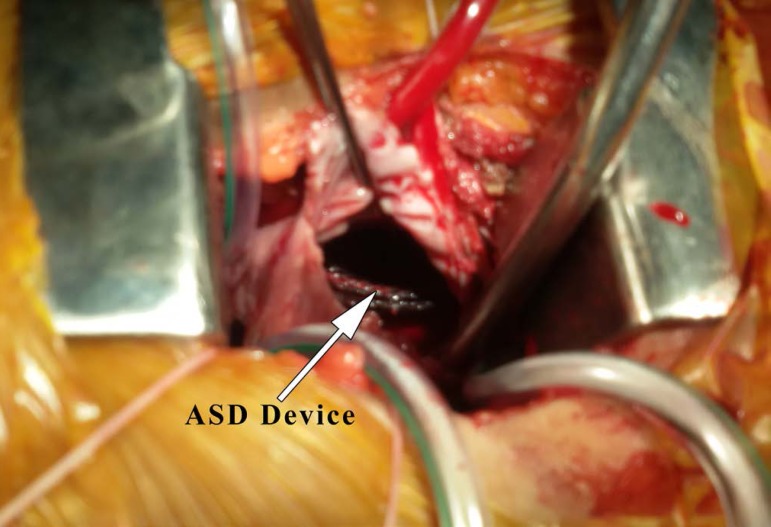
Migrated ASD closure device in right atrium seen preoperatively on minithoracotomy approach.

In the patients undergoing right minithoracotomy, the device was found in the right atrium, (Figure 4), in six patients and in left atrium in two patients, one impinged in the tricuspid valve, two in the right ventricle and one in the main pulmonary artery (Table 2).

**Table 2:** Operative details of all patients undergoing device failure.

Anterior minithoracotomy																			
S.N	Weight (kg)	Age	Sex	ASD device size	CPB Time	AOX Time	Chest Drainage	Femoral artery cannula size	Femoral Venous size	ASD size on echocardiography		PAH	Closure Technique		H.S (Days)	V.T (Hours)	Site of Migration	Deficient of Margin	Reason for sternotomy
1	37	41	0	30	35	15	250	18	24/29	24		2	Direct		10	4	RA	IVC	
2	44	65	0	32	33	14	0	16	24/29	28		1	PTFE		5	4	LA	IVC	
3	37	30	1	28	98	48	0	18	24/18	24		1	PTFE		9	3	RV	-	
4	60	47	1	36	64	29	0	20	24/29	32		2	PTFE		8	3	RA	IVC	
5	65	42	0	32	60	18	0	18	24/29	28		1	PTFE		12	2	TV	IVC	
6	13	5	1	36	44	19	0	12	14/18	30		1	PTFE		5	3	RA	IVC	
7	53	45	1	28	50	21	0	16	24/26	24		2	Pericardial		7	3	RA	-	
8	58	25	0	32	57	23	50	16	24/29	26		1	PTFE		4	3	RA	-	
9	39	15	0	24	59	22	0	16	20	20		1	PTFE		5	3	LA	-	
10	52	40	0	24	58	29	150	18	24/26	18		1	Direct		8	3	RV	-	
11	39	28	0	40	32	16	100	20	22/28	36		1	PTFE		5	3	RA	IVC	
12	27	12	0	28	98	81	0	14	20	24		1	Pericardial		8	3	MPA	-	
Midline sternotomy																			
13	42	15	1	28	50	20	100			24	1		PTFE	6		3	MPA		Cardiac Tamponade
14	50	38	0	24	58	22	150			18	1		Direct	6		6	LPA		Inaccessibility
15	30	12	0	24	78	45	0			20	1		Direct	8		3	LVOT		Hemodynamic instability

H.S=Hospital Stay; V.T=ventilator time; R.A=right atrium; LA=left atrium; MPA=main pulmonary artery; LPA=left pulmonary artery; TV=tricuspid valve; R.V=Right Ventricle; IVC=Inferior vena cava; LVOT=left ventricular outflow tract; PAH=pulmonary arterial hypertension (1=Mild; 2=Moderate); AOX=aortic cross clamp

In the remaining three patients, sternotomy was performed due to hemodynamic instability in two patients and device migration into left pulmonary artery (LPA) (unapproachable site through anterior minithoracotomy) in one patient. The two patients with hemodynamic instability had cardiac tamponade due to rent in right atrium which was repaired intraoperatively. The device migrated into LVOT was retrieved through left atrium and the device migrated to LPA was retrieved through pulmonary arteriotomy.

Among the 15 patients with device migration, six had deficient inferior vena cava margin, five patients had flimsy margin and one had deficient superior margin. In the patient with device migration into LVOT, two ASD with thin band of tissue between them was observed.

Aortic cross-clamp time average was 22 minutes (range 14 to 51 minutes) while mean CPB time was 58.27±20.35 minutes (range 32 to 98 minutes). No patient required reoperation for bleeding or surgical complication. No operative or in-hospital mortality was observed. No patient developed wound related, vascular or neurological complications. One patient who had hemodynamic instability preoperatively developed renal dysfunction that recovered spontaneously. Postoperative echocardiography showed adequately closed ASD without mitral or tricuspid regurgitation and ventricular dysfunction in all the patients.

The mean follow-up period was six months. All patients in the follow-up were in New York Heart Association class I without symptoms in the last follow-up. Echocardiography during follow-up period confirmed absence of any residual defect with normal ventricular function.

## DISCUSSION

The risk of complications following ASD device closure is less than 1% with the use of presently available septal occluders. The common complications are device malposition, perforation, embolization, thrombosis, residual shunt and infective endocarditis. Some surgeons device embolism reported as frequent complication after ASD device closure ranging from 5%-20% with different devices and series^[4,9]^. A survey on AGA Medical Corporation (Plymouth, MN, USA) proctors, conducted in 2004, determined the rate of embolization to be 21 of 3824 implants (0.55%)^[10]^. In a series by Chessa et al.^[11]^, among 417 patients with ASD who underwent catheter closure of ASD, 258 received Amplatzer septal occluder devices. Complication rate in their series was 8.65% with device embolization/malposition as the most common complication occurring in 3.5% cases. Total 2/3 (2.5%) patients required surgical retrieval.

The factors implicated in device embolization have been larger ASD (> 20 mm) and device size (> 24 mm), under device sizing, inadequate atrial rim or thin ASD rim^[12]^. In this series, all patients had large ASDs with six having deficient inferior rim and five had margins made of thin flimsy fenestrated tissue (one patient had with two ASDs separated by thin tissue) and ASD of one had with deficient superior rim. Appropriate patient selection and an accurate device selection is mandatory to prevent serious complications such as ventricular arrhythmias, outflow tract obstruction of the left and right ventricle, or ischemic events secondary to the obstruction of blood flow due to device embolization^[13]^.

In an analysis by DiBardino et al.^[10]^, since July 1, 2002, 223 adverse events in patients undergoing Amplatzer ASD closure were submitted to the Food and Drug Administration, resulting in 17 (7.6%) deaths and 152 (68.2%) surgical rescue operations. The mortality for surgical management of a device adverse event (2.6%) was 20-fold higher than for primary elective ASD closure (0.13%, *P*<0.0001)^[10]^.

In one of the patients with device migration into LVOT, two ASDs with a thin band of tissue between them were identified, which probably led to misinterpretation and the use of a smaller device leading to its migration into LVOT.

One of the patients operated through minithoracotomy with device into main pulmonary artery had longer CPB time due to impingement of the device in pulmonary artery, and it was carefully removed avoiding injury to pulmonary valve.

In the last two decades, mini-invasive closure of ASD has become a preferred procedure with equally good surgical results and better cosmetic results^[14-16]^ compared to midline sternotomy. At our institute, all ASD patients were operated through right anterior minithoracotomy with peripheral CPB in adults and central cannulation in small children (10 kg). However, midline sternotomy still remains a standard approach for patients requiring surgery for complication following device closure. These patients lose the cosmetic and sternal sparing advantage of mini-invasive approach. In patients with device embolization and perforation leading to tamponade or LVOT or RVOT obstruction or any other complication requiring emergent surgical intervention^[17]^, midline sternotomy has advantage over mini-invasive approach, therefore it is rapid, gives full access to all cardiac chambers and great vessels, making it easier to manage the complications. However, in selected patients with device migration without hemodynamic instability or any severe complication, patients can be operated through minithoracotomy approach with femoral vessel cannulation for CPB without increasing the risk of cardiac, vascular or neurological complications as shown by our results. In our series, 85% of the patients, when referred, were hemodynamically stable without any other complication (apart from device migration). All these patients were successfully operated through minithoracotomy without the need for conversion to sternotomy. Femoral artery and femoral vein cannulation were used in all our patients for CPB. Device was easily retrieved without injury to mitral or tricuspid valve. Both aortic cross clamp time and CPB time were comparable to other study on mini-invasive approach^[18]^.

We believe that there are certain contraindications to mini-invasive approach in patients with complication following *ostium secundum* ASD device closure. The most important contraindication is presence of hemodynamic instability either due to left ventricular outflow obstruction, cardiac perforation with tamponade or migration into ascending aorta or arch. Other contraindications are migration of device into branch pulmonary artery and mitral or tricuspid valve entrapment. Although, device can be retrieved from branch pulmonary artery through this approach, consequently, sternotomy is believed to be safer in this subset of patients.

## CONCLUSION

In our experience, minithoracotomy with femoral cannulation for CPB is a safe approach for selected group of hemodynamicaly stable patients with device migration into approachable locations following transcatheter device closure of ASD without increasing the risk of cardiac, vascular or neurological complications with good cosmetic and surgical results.

**Table:** 

Authors' roles & responsibilities	
VW	Substantial contributions to the conception or design of the work; or the acquisition, analysis, or interpretation of data for the work; drafting the work or revising it critically for important intellectual content; final approval of the version to be published
CD	Agreement to be accountable for all aspects of the work in ensuring that questions related to the accuracy or integrity of any part of the work are appropriately investigated and resolved; final approval of the version to be published
MH	Drafting the work or revising it critically for important intellectual content; final approval of the version to be published
PG	Final approval of the version to be published
KP	Final approval of the version to be published
AM	Drafting the work or revising it critically for important intellectual content; final approval of the version to be published
PS	Substantial contributions to the conception or design of the work; or the acquisition, analysis, or interpretation of data for the work; final approval of the version to be published
